# Rapid Drug Sensitivity Profiling via a Novel High-Success-Rate Culture Method for Patient-Derived Pancreatic Cancer: An Exploratory Preclinical Platform for Advancing Clinical Applications and Drug Development

**DOI:** 10.3390/cells15040313

**Published:** 2026-02-07

**Authors:** Yu Kato, Naoki Yamamoto, Yuichiro Uchida, Noriko Hiramatsu, Takato Ozeki, Yukari Minobe, Yukika Hasegawa, Sho Kawabe, Hikaru Yabuuchi, Seiji Yamada, Yuko Hata, Eiji Sugihara, Tetsuya Takimoto, Kuniaki Saito, Takeshi Takahara, Koichi Suda, Osamu Nagano, Hideyuki Saya

**Affiliations:** 1 Oncology Innovation Center, Research Promotion Headquarters, Fujita Health University, Aichi 470-1192, Japan; yu.toki@fujita-hu.ac.jp (Y.K.); norikoh@fujita-hu.ac.jp (N.H.); yamadas@fujita-hu.ac.jp (S.Y.); eiji.sugihara@fujita-hu.ac.jp (E.S.); tetsuya.takimoto@fujita-hu.ac.jp (T.T.); osamu.nagano@fujita-hu.ac.jp (O.N.); hsaya@fujita-hu.ac.jp (H.S.); 2 Center for Society-Academia Collaboration, Research Promotion Headquarters, Fujita Health University, Aichi 470-1192, Japan; takato.ozeki@fujita-hu.ac.jp (T.O.); yukari.minobe@fujita-hu.ac.jp (Y.M.); yukika@fujita-hu.ac.jp (Y.H.); sho.kawabe@fujita-hu.ac.jp (S.K.); hikaru.yabuuchi@fujita-hu.ac.jp (H.Y.); saitok@fujita-hu.ac.jp (K.S.); 3 Graduate School of Health Sciences, Fujita Health University, Aichi 470-1192, Japan; 4 International Center for Cell and Gene Therapy, Research Promotion Headquarters, Fujita Health University, Aichi 470-1192, Japan; 5 Department of Pharmaceutical Engineering, Faculty of Engineering, Sanyo-Onoda City University, Yamaguchi 756-0884, Japan; 6 Department of Surgery, School of Medicine, Fujita Health University, Aichi 470-1192, Japan; yuichiro.uchida3389@gmail.com (Y.U.); takahara731026@yahoo.co.jp (T.T.); ko-suda@nifty.com (K.S.); 7 Clinical Laboratory, Fujita Health University Hospital, Aichi 470-1192, Japan; 8 Open Facility Center, Research Promotion Headquarters, Fujita Health University, Aichi 470-1192, Japan; yuko.hata@fujita-hu.ac.jp

**Keywords:** pancreatic ductal adenocarcinoma, patient-derived cells, primary new culture method, drug sensitivity assay, *KRAS* mutation, functional precision medicine, fibroblast activation protein

## Abstract

**Highlights:**

**What are the main findings?**

- A cost-effective, hydrogel-free adherent culture system for patient-derived pancreatic cancer cells was established with a high success rate exceeding 90%.
- The cultured cells maintain essential oncogenic *KRAS* mutations and exhibit a consistent six-gene malignancy signature, including fibroblast activation protein (*FAP*) and *WNT5A*.

**What are the implication of the main findings?**

- This platform enables rapid and accurate drug sensitivity profiling that shows potential associations with clinical trends, serving as a practical tool for functional precision medicine.
- The high-purity cancer cell population supports an exploratory preclinical platform and biomarker validation for novel therapeutics, such as antibody–drug conjugates and immune checkpoint inhibitors.

**Abstract:**

Pancreatic cancer is a highly intractable malignancy that necessitates personalized treatment strategies. Conventional patient-derived models, such as three-dimensional organoids, are often limited by intellectual property constraints and high costs. In this study, we developed an affordable adherent culture system for patient-derived pancreatic cancer cells using a proprietary medium and laminin-coated dishes. Primary cultures were successfully established from 28 patients with pancreatic ductal adenocarcinoma, exceeding a 90% success rate. Validation of eight samples confirmed maintenance of epithelial cell adhesion molecule expression and preservation of oncogenic *KRAS* mutations. Transcriptomic profiling revealed consistent upregulation of a six-gene signature (*FAP*, *IGFBP5*, *PRRX1*, *SPARC*, *WNT5A*, and *ADAMTS12*), which is associated with malignancy. In vitro drug sensitivity assays revealed interpatient heterogeneity with preliminary clinical associations. In conclusion, this simplified platform provides high-purity cancer cells and serves as a functional precision medicine tool. Beyond conventional chemotherapy, this platform has the potential to support applications ranging from biomarker validation and exploratory preclinical testing of novel therapeutics, including immune checkpoint inhibitors and antibody–drug conjugates. This optimization can lead to personalized therapeutic strategies for pancreatic cancer.

## 1. Introduction

Pancreatic cancer remains one of the most intractable malignancies in Japan, with a 5-year relative survival rate of approximately 12.7% [1]. Even for patients diagnosed and resected at Stage I, the 5-year survival rate remains as low as 40–50%, highlighting the challenge of preventing postoperative recurrence [1]. Currently, while tens of thousands of cancer genomic profiling tests are performed annually in Japan, the rate of patients who eventually receive genome-matched therapy remains at approximately 10% [2,3]. Furthermore, nearly 30% of cases are reported as “inconclusive” or fail to complete the analysis due to insufficient sample quality or quantity [2,4]. Under the Japanese national health insurance system, cancer genomic profiling is limited to a single occurrence per lifetime, making it difficult to monitor clonal evolution or reevaluate genomic profiles at the time of recurrence. Since standard chemotherapy regimens are largely protocolized by cancer type, there is a significant unmet medical need for a functional evaluation system using high-purity patient-derived cancer cells to select the most effective agents from the early stages of treatment.

Currently, organoid culture is the predominant method for expanding patient-derived cancer cells. This technique involves culturing cells in a three-dimensional (3D) environment using extracellular matrix-mimicking hydrogels, such as Matrigel, to recapitulate the structural and functional characteristics of the original tumor [5,6].

However, organoid-related technologies are protected by extensive patent portfolios, often requiring complex and costly licensing agreements for commercial use, industry-academic collaborations, or high-throughput drug screening [7,8,9]. In addition to these intellectual property constraints, operational challenges remain, including the high cost and batch-to-batch variability of hydrogels, as well as the difficulty in suppressing the overgrowth of stromal components, such as fibroblasts and vascular endothelial cells [10].

KRAS mutations are the primary driver in pancreatic ductal adenocarcinoma (PDAC), occurring in more than 90–95% of cases [11,12]. Among these, substitutions at codon 12 are the most prevalent, specifically G12D (approx. 40–44%), G12V (approx. 30–34%), and G12R (approx. 15–20%) [13]. These mutations play a central role in tumor initiation, progression, and resistance to therapy, making them essential molecular markers for confirming the identity and purity of patient-derived cancer cells.

Pancreatic cancer has a poor prognosis primarily due to delayed diagnosis and high metastatic potential. Exosomes, particularly exosome-derived microRNAs, have emerged as promising non-invasive biomarkers; however, the search for additional useful markers remains essential [14].

The objective of this study was to establish a novel, cost-effective, and simplified adherent culture system for patient-derived pancreatic cancer cells based on our patented technology, which eliminates the need for an extracellular matrix. This method not only overcomes the intellectual property constraints and operational complexities associated with conventional organoid cultures but also enables the rapid expansion of high-purity cancer cells by suppressing stromal contamination [10]. We validated the reliability of this culture system through KRAS mutation analysis using highly sensitive droplet digital polymerase chain reaction (ddPCR) and comprehensive transcriptomic profiling via RNA sequencing (RNA-seq). Furthermore, we demonstrated its clinical utility by performing in vitro drug sensitivity assays for major chemotherapeutic agents. Our findings suggest that this technology can serve as a functional precision medicine platform, complementing genomic profiling and contributing to the optimization of personalized treatment strategies for pancreatic cancer.

## 2. Materials and Methods

### 2.1. Patients and Clinical Samples

Tissue samples were obtained from eight patients with invasive PDAC who underwent surgical resection at Fujita Health University Hospital between November 2024 and October 2025 (Table 1). Patients who had received neoadjuvant chemotherapy or radiotherapy were included to evaluate their potential impact on primary cancer cell culture. During this period, tissue samples were collected from 28 patients for culture.

This study was conducted in accordance with the Declaration of Helsinki and approved by the Medical Research Ethics Committee of Fujita Health University (Approval No. HG23-008 and HM25-012). Written informed consent for the use of clinical samples and data for research purposes was obtained from all patients prior to surgery.

Immediately following surgical resection, tumor (T) and non-tumor (N) tissue specimens (approximately 5 mm in size) were collected, placed in culture medium supplemented with a storage solution and antibiotics (Penicillin–Streptomycin; FUJIFILM Wako Pure Chemical Corp., Osaka, Japan), and transported to the laboratory within 1 h while maintaining the temperature between 15–20 °C.

### 2.2. Primary Cell Culture

Tissue specimens were enzymatically dissociated using collagenase (Sigma-Aldrich, St. Louis, MO, USA) with agitation at 37 °C for 20 min [15]. Following centrifugation and washing, cells were cultured using a newly developed proprietary medium (Patent No. 7735022, PCT/JP2024/046241). Unlike conventional 3D organoid culture methods, this system does not require embedding cells in an extracellular matrix. Instead, we utilized a unique adherent culture technique where cells were seeded onto laminin-coated dishes (Fujifilm Wako). PDAC and other cancer cells exist within a laminin-rich basement membrane. Laminin enables cells to recognize their polarity toward the basement membrane, facilitating the maintenance of epithelial cell properties and stabilization of proliferation. This culture method selectively promotes the adhesion and proliferation of epithelial cell adhesion molecule (EpCAM)-positive cells, which are highly expressed on the surface of most cancer cells, including pancreatic cancer cells. Consequently, the overgrowth of stromal components, such as vascular endothelial cells and lymphocytes, is naturally suppressed without the need for additional cell-sorting procedures. Subculturing was performed using standard trypsinization (TrypLE™ Select Enzyme, Thermo Fisher Scientific Inc., Waltham, MA, USA) within a treatment time of 10 min.

### 2.3. Morphology and Immunohistochemical Staining

Formalin-fixed paraffin-embedded (FFPE) sections were prepared from paraffin blocks used for pathological diagnosis of the target cases. Hematoxylin and eosin staining was performed using standard protocols, and representative images were captured with an upright microscope (BX-51; Olympus, Tokyo, Japan). The morphology of the cultured cells was routinely monitored and imaged using an inverted microscope (IX-71; Olympus). As a control, the human pancreatic cancer cell line AsPC-1 (ATCC CRL-1682) was utilized.

Cultured cells were prepared into cell smears using a Cytospin™ 4 centrifuge (Epredia, LLC, Kalamazoo, MI, USA) at cell passage, stained with anti-human EpCAM rabbit antibody (Cell Signaling Technology, Inc. Danvers, MA, USA), and imaged under a fluorescence microscope (BX-51, Olympus) using anti-rabbit Alexa488-labeled antibody (Thermo Fisher Scientific) as the secondary antibody. Nuclei were counterstained with DAPI.

To validate the expression and localization of fibroblast activation protein alpha (FAP), immunohistochemical (IHC) staining was performed using FFPE sections from both T and adjacent N tissues. The sections were subjected to heat-induced antigen retrieval in Tris-EDTA buffer (pH 9.0) at 95 °C for 40 min. To minimize non-specific binding, sections were incubated with a protein blocking reagent (X0909, Dako; Agilent Technologies, Inc., Santa Clara, CA, USA). The primary antibody against FAP (Proteintech Group, Inc., Rosemont, IL, USA) was applied at a dilution of 1:200 and incubated for 1 h at 37 °C. After washing with phosphate-buffered saline, sections were incubated with a secondary anti-rabbit antibody polymer reagent (Nichirei Biosciences Inc., Tokyo, Japan) for 1 h at 37 °C. Following an additional phosphate-buffered saline wash, the signal was visualized using 3,3′-diaminobenzidine (chromogen, Agilent Technologies), and nuclei were counterstained with hematoxylin, the dehydrated, cleared, and mounted for microscopic observation.

### 2.4. Genomic DNA Extraction and KRAS Genotyping by Droplet Digital PCR

Genomic DNA was extracted from cultured cells (3rd or 4th passage) using Maxwell^®^ RSC Cultured Cell DNA Kit (Cat No. AS1620; Promega, Madison, WI, USA) with a Maxwell^®^ RSC Instrument according to the manufacturer’s instructions. DNA concentration was quantified using a Quantus Fluorometer with the QuantiFluor dsDNA System (Promega).

The ddPCR reaction for KRAS G12R or G12V had a total volume of 25 µL, comprising 0.25 µM dPCR probes (FAM: KRAS G12R or G12V, HEX: KRAS exon 2 wild-type, Supplementary Table S1), 0.9 µM of forward and reverse primers, 5 µL DNA template, and ddPCR Multiplex Supermix (#12005909, Bio-Rad Laboratories, Inc., Hercules, CA, USA), with the reaction brought to final volume using ddH_2_O. The ddPCR reaction for KRAS G12D had a total volume of 25 µL, comprising ddPCR Supermix for Probes, ddPCR Mutation Assay: KRAS p.G12D (#1863506, Multiplex Mutation Screening Kits, Bio-Rad Laboratories), and 5 µL DNA template, with the reaction brought to final volume using ddH_2_O.

Droplets were generated using the QX200™ Droplet Digital™ PCR System (#1864001, Bio-Rad Laboratories) and transferred to a 96-well PCR plate using the droplet generator. After sealing, the plate was subjected to PCR amplification. The PCR conditions were as follows: pre-denaturation at 95 °C for 10 min, followed by 40 cycles of 94 °C for 30 s and 60 °C (G12R, G12V) or 55 °C (G12D) for 60 s, with final inactivation at 98 °C for 10 min, at a heating rate of 2 °C/s. After amplification, droplet fluorescence signals were captured using a QX200 droplet reader and analyzed using QuantaSoft™ software (Version 1.7.4.0917, Bio-Rad Laboratories). For interpretation of ddPCR results, a threshold of ≥1.0% mutant allele frequency was established to determine mutation positivity. This stringent cutoff was applied to ensure the reliable identification of cancer-derived genomic variants while minimizing potential background noise.

### 2.5. In Vitro Chemotherapy Drug Sensitivity Assay

Chemosensitivity of patient-derived pancreatic cancer cells (3rd or 4th passage) to gemcitabine hydrochloride (gemcitabine), paclitaxel (Fujifilm wako), and SN-38 (Tokyo Chemical Industry Co., Ltd., Tokyo, Japan) was evaluated. Cells were seeded into 96-well plates at a density of 5 × 10^3^ cells/well using the patented culture method and allowed to adhere overnight. They were then treated with varying concentrations of each drug: gemcitabine (0.5 µM to 100 µM), paclitaxel (5 nM to 1000 nM), and SN-38 (5 nM to 1000 nM).

After incubating for 48 h, cell viability was measured using CCK-8 assay (Dojindo Molecular Technologies, Inc., Kumamoto, Japan) following the manufacturer’s instructions. Absorbance was recorded with a Multiskan™ FC microplate reader (Thermo Fisher Scientific). The half-maximal effective concentration (*EC_50_*) was calculated from the dose–response curves using Microsoft Excel (Version 365, Microsoft Corporation, Redmond, WA, USA). All experiments were performed in triplicate.

### 2.6. RNA Extraction and Next-Generation Sequencing

Total RNA was extracted from CRL-1682 and each cultured cell sample (3rd or 4th passage) using an RNA extraction kit (RNeasy Mini Kit, QIAGEN GmbH, Hilden, Germany). The concentration and purity of the extracted RNA were measured using a NanoDrop spectrophotometer (Thermo Fisher Scientific). To ensure high-quality sequencing data, RNA integrity was assessed using a TapeStation (Agilent Technologies, Inc., Santa Clara, CA, USA), and only samples with an RNA integrity number ≥ 7.0 were utilized for subsequent analysis.

RNA-seq libraries were prepared using the NEBNext Poly(A) mRNA Magnetic Isolation Module and the NEBNext Ultra II Directional RNA Library Prep Kit for Illumina (New England Biolabs, Inc., Ipswich, MA, USA) according to the manufacturer’s instructions. Sequencing was performed on the NextSeq 2000 system (Illumina, Inc., San Diego, CA, USA) using the NextSeq 1000/2000 P2 XLEAP-SBS Reagent Kit (Cat. No. 20100987).

### 2.7. Bioinformatics and Pathway Analysis and Statistical Processing

Differentially expressed genes (DEGs) were identified by comparing expression profiles between N and T samples from the same patient (PK-56N vs. PK-56T), as well as across all N and T samples (PK-All(N) vs. PK-All(T)). DEGs were defined as genes with log_2_ fold change ≥ 1.0 and a false discovery rate (FDR) < 0.05 (Fold Change > 2.0 and Adjusted *p*-value < 0.05), and only genes upregulated in tumor samples were retained for downstream analyses. The Benjamini–Hochberg method was used for multiple hypothesis testing to control FDR. RNA-seq data analysis was performed using CLC Genomics Workbench ver. 25.0.3. (QIAGEN GmbH).

For the common DEGs identified in both comparisons, Gene Ontology (GO) and Kyoto Encyclopedia of Genes and Genomes (KEGG) pathway enrichment analyses were performed using DAVID (DAVID 2021, DAVID Knowledgebase v2023q4) [16,17]. Furthermore, the clinical and biological relevance of candidate genes was cross-referenced with the GeneCards database [18,19], with a particular focus on genes previously reported to be associated with pancreatic pathophysiology.

### 2.8. Quantitative Real-Time PCR Verification of RNA-Seq Data

To validate candidate genes identified from RNA-seq analysis, quantitative reverse transcription-PCR (qRT-PCR) was performed. Total RNA, identical to that used for RNA-seq, was reverse-transcribed into cDNA using the reagents included in the TaqMan™ Gene Expression Cells-to-CT™ Kit (Thermo Fisher Scientific). qPCR assays were conducted on a QuantStudio 1 Real-Time PCR System (Thermo Fisher Scientific) using TaqMan™ Gene Expression Master Mix (Thermo Fisher Scientific) and specific TaqMan™ Gene Expression Assays (TaqMan Primers and Probes, Thermo Fisher Scientific). Details of TaqMan primers and probes used in this study are shown in Table 2. Target gene expression levels were normalized to *ACTB* (actin beta) as an endogenous control. Relative expression levels were calculated using the ΔΔ*Ct* method, with the expression level of PK-56N serving as the reference (control).

## 3. Results

### 3.1. Histopathological Characterization of Pancreatic Tissue Samples

**Figure 1:**
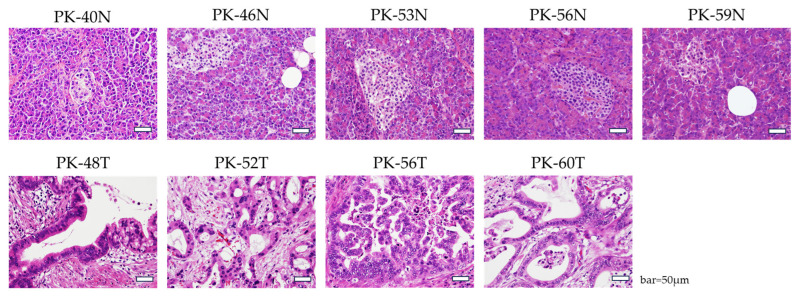
Histopathological features of pancreatic ductal adenocarcinoma tumor (T) tissues and adjacent non-tumor (N) tissues. Representative hematoxylin and eosin staining of patient-derived pancreatic tissues. (**Upper panels**): adjacent N tissues showing normal acinar architecture and pancreatic islets. (**Lower panels**): T tissues showing typical features of PDAC, characterized by irregular glandular structures and dense desmoplastic stroma. Scale bars = 50 μm.

Hematoxylin and eosin staining was performed to evaluate the histopathological features of the N and T tissues (Figure 1). N sections showed well-organized acinar structures and islets of Langerhans, consistent with normal pancreatic morphology. T sections showed characteristic features of PDAC, including irregular ductal structures composed of atypical cells and prominent desmoplastic stroma. These findings confirmed the identity and purity of tissues used for subsequent primary cell culture and molecular analyses.

### 3.2. Morphological Characterization and Epithelial Marker Expression in Established Primary Cells

**Figure 2:**
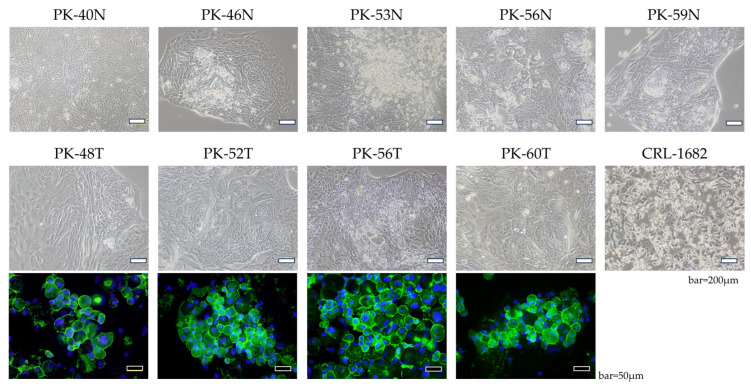
Morphological characteristics and epithelial marker expression of established pancreatic cells. (**Upper and middle panels**): representative phase-contrast images of primary adherent cultures derived from N and T pancreatic tissues, compared with the CRL-1682 cell line. Scale bars = 200 μm. (**Lower panels**): immunofluorescence staining for EpCAM (green) in tumor-derived cells. Nuclei were counterstained with DAPI (blue). Scale bars = 50 μm.

During the study period, cancer cell cultures were successfully established in 26 of 28 cases (92.9%). In cases where the culture was unsuccessful, examination of tissue specimens prepared simultaneously confirmed that the excised tissue contained only a very small number of cancer cells. Phase-contrast microscopy revealed that primary cells derived from both N and T tissues exhibited a typical epithelial-like, cobblestone morphology (Figure 2). To further confirm their epithelial origin, we performed immunofluorescence staining for EpCAM, a specific marker for epithelial cells. All tested tumor-derived cell lines showed robust membranous expression of EpCAM (Figure 2, lower panels), confirming the epithelial origin of the cultured cells. These findings demonstrate that the established primary cultures maintain their epithelial characteristics. In this study, primary cultures were successfully established in all cases examined for both N and T tissue sections. For reference, the developed culture method has been applied to over 28 pancreatic cancer cases to date, achieving success in primary culture in over 90% of them.

### 3.3. Validation of KRAS Mutation Status via ddPCR

To confirm the presence of KRAS driver mutations and ensure the purity of clinical samples, we performed ddPCR targeting the most prevalent KRAS exon 2 mutations (G12D, G12V, and G12R). In T samples, oncogenic KRAS mutations were successfully identified in all cases, with mutant allele frequency ranging from 1.3% to 38.6%. The 1D droplet amplitude plots for T samples showed distinct clusters of mutant-positive droplets (FAM-positive) and wild-type-positive droplets (HEX-positive), allowing for precise quantification of the mutant allele fraction.

**Figure 3:**
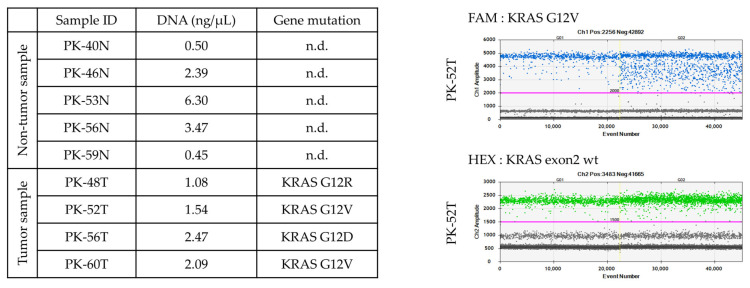
KRAS mutation status in patient-matched pancreatic N and T samples. (**Left**): table summarizing the KRAS genotype and DNA concentration for each sample. Oncogenic KRAS mutations (G12D, G12V, or G12R) were identified in T samples, whereas no mutations (n.d., not detected) were found in the corresponding adjacent N tissues. (**Right**): representative 1D droplet amplitude plots for sample PK-52T. The **upper** plot (FAM channel) shows a distinct cluster of positive droplets for the KRAS G12V mutation, and the **lower** plot (HEX channel) shows positive droplets for the KRAS exon 2 wild-type (wt) sequence.

In all corresponding N samples, no mutant-positive droplets were detected, while wild-type signals remained consistent with the total DNA input. This complete absence of mutant droplets in N samples validated the high specificity of our sampling process and confirmed that the adjacent “normal” tissues were free from detectable tumor cell infiltration at the genomic level (Figure 3).

### 3.4. In Vitro Drug Sensitivity and Clinical Clinicopathological Correlations

**Figure 4:**
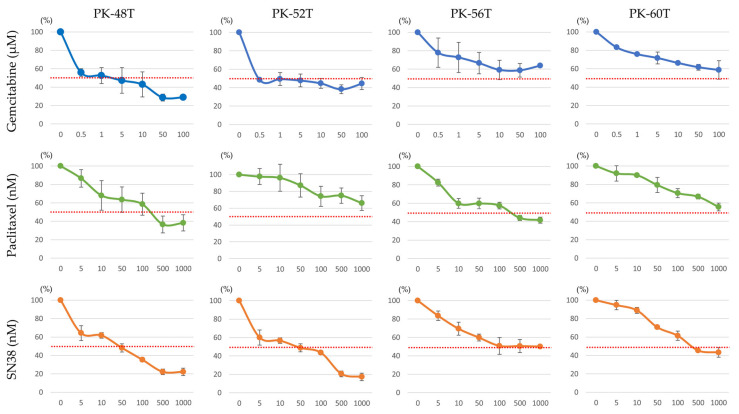
In vitro drug sensitivity of gemcitabine, paclitaxel, and SN38. The horizontal axis represents drug concentration; the vertical axis represents cell viability normalized to control WST-8 absorbance. Microsoft Excel was used to calculate the mean and standard deviation. The red dashed line indicates the *EC*_50_ cell activity.

We evaluated the chemosensitivity of the established primary cells to standard chemotherapeutic agents, including gemcitabine, paclitaxel, and SN38 (Table 3). The drug response profiles exhibited significant interpatient heterogeneity. For instance, PK-52T demonstrated high sensitivity to gemcitabine (*EC*_50_ = 0.5 µM), whereas PK-56T and PK-60T displayed marked resistance (*EC*_50_ > 100 µM). A similar trend of differential sensitivity was observed for SN38, particularly in PK-56T (Figure 4).

The in vitro drug sensitivity profiles appeared to correlate with clinical outcomes; the gemcitabine-resistant line PK-56T originated from a poorly differentiated tumor with recurrence < 6 months post-resection. These data suggest our patient-derived cell models preserve the functional characteristics and therapeutic vulnerabilities of the original tumors.

The clinical cases of PK-46 and PK-60 illustrate potential associations of our model with clinical outcomes. PK-46 experienced significant adverse events during neoadjuvant chemotherapy (NAC), with CA19-9 levels showing an incomplete response (baseline: 2653 U/mL; day 35: 1109.7 U/mL, still markedly elevated). Despite a prolonged NAC period of 282 days, PK-60’s CA1-9 level increased from 7 U/mL to 76.1 U/mL by the end of treatment. We observed a pattern where patients with high in vitro drug resistance were associated with recurrence or elevated CA19-9 levels. The results suggest our primary culture-based drug sensitivity testing may serve as a valuable diagnostic tool for predicting therapeutic responses and prognosis, which could facilitate selection of optimal treatment strategies for patients with PDAC.

### 3.5. Differential Gene Expression in Paired and Aggregate Samples

**Figure 5:**
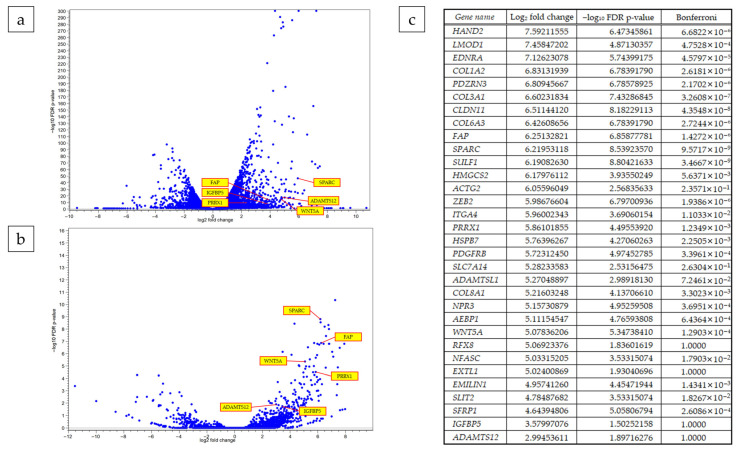
Differential gene expression analysis between N and T samples. Volcano plots illustrating differentially expressed genes in (**a**) a representative patient-matched pair (PK-56N vs. PK-56T) and (**b**) all pooled N vs. T samples (PK-All(N) vs. PK-All(T)). (**c**) List of representative genes consistently up FAP regulated in both the PK-56 pair and the aggregate analysis, including oncogenic and stromal-related markers such as *FAP*, *SPARC*, *WNT5A*, and *PRRX1*.

To elucidate the molecular characteristics of cells established via our adherent culture method, we performed RNA-seq on both patient-matched pairs (T vs. N) and an aggregate cohort. The volcano plot for a representative pair, PK-56N vs. PK-56T, revealed a robust differential expression profile, with significant upregulation of genes associated with tumor progression and inflammation, such as *SERPINA3*, *DUOX2*, and *LCN2*. Several collagen family genes (e.g., *COL1A2*, *COL3A1*) were significantly enriched in the tumor-derived cells, consistent with the desmoplastic nature often preserved in pancreatic cancer models (Figure 5a).

The aggregate analysis (PK-All(N) vs. PK-All(T)) further validated these findings, demonstrating consistent transcriptomic shifts across the cohort. In the aggregate volcano plot, we observed marked upregulation of *HSPB6*, *SPARC*, and *SULF1*, which are known to be associated with extracellular matrix remodeling and tumor–stroma interactions. Conversely, genes such as *GKN1* and *CPA2* showed significant downregulation in the tumor group. The high −log_10_ FDR *p*-values and the wide distribution of log_2_ fold changes underscore the high signal-to-noise ratio and the distinct biological identity of the cells established by our culture protocol (Figure 5b). We then examined the genes that were elevated in PK-56T vs. PK-56N and in PK-All(T) vs. PK-All(N), identifying 30 genes (Figure 5c). These DEGs provided a highly filtered and reliable gene list for subsequent functional enrichment analysis.

### 3.6. Integrative Functional and Pathway Analysis

Functional enrichment analysis was performed using the 30 genes consistently upregulated across both paired and aggregate comparisons. To systematically characterize the biological programs activated in the tumor-derived cells, we performed an integrated functional enrichment analysis using DAVID, incorporating both GO and KEGG pathway databases.

The DEGs showed prominent enrichment in biological processes essential for tumor malignancy. GO analysis revealed highly significant clusters related to angiogenesis (*p* = 1.32 × 10^−5^) and vasculature development (*p* = 1.01 × 10^−7^). Furthermore, terms associated with active cellular movement, such as cell migration (*p* = 7.05 × 10^−6^ and cell motility (*p* = 4.11 × 10^−5^), were markedly overrepresented in tumor-derived cells compared to their non-tumor counterparts.

KEGG pathway analysis further elucidated the molecular networks driving these phenotypic shifts. We identified significant enrichment in pathways critical for cell–substrate interaction and survival, including focal adhesion (*p* = 0.00705) and extracellular matrix–receptor interaction (*p* = 0.0132). The PI3K-Akt signaling pathway, a master regulator of cell growth and survival in pancreatic cancer was significantly enriched (*p* = 0.0331), along with cell adhesion molecules (*p* = 0.0386). The convergence of structural remodeling processes (extracellular matrix interaction and focal adhesion) with pro-survival signaling (PI3K-Akt) and morphogenetic programs (angiogenesis) strongly suggests that our adherent culture model successfully recaptures the invasive and adaptive transcriptomic landscape of pancreatic adenocarcinoma (Supplementary Table S2).

### 3.7. Validation of Differentially Expressed Genes via qRT-PCR

To validate the reliability of our RNA-seq findings, we performed qRT-PCR on six representative genes: *FAP*, *IGFBP5*, *PRRX1*, *SPARC*, *WNT5A*, and *ADAMTS12*.

**Figure 6:**
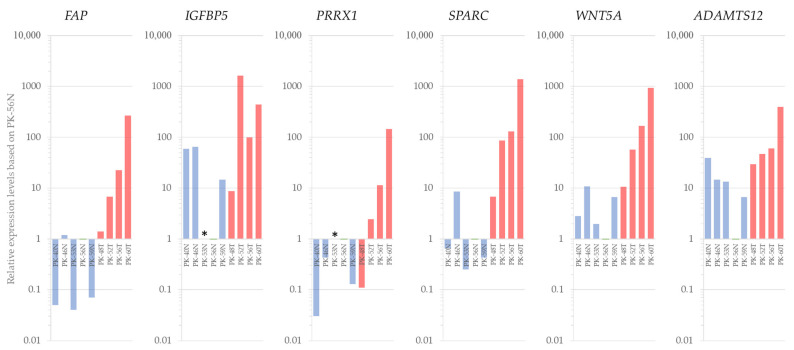
Validation of differentially expressed genes by qRT-PCR. Relative mRNA expression levels of six candidate genes (*FAP*, *IGFBP5*, *PRRX1*, *SPARC*, *WNT5A*, and *ADAMTS12*) were analyzed in patient-matched N- (blue bars) and T-derived cells (red bars). All data are presented as relative expression levels normalized to the PK-56N sample (set as 1.0, green bars). Asterisks (*) indicate samples where the target gene expression was below the limit of detection.

The qRT-PCR results were highly consistent with the transcriptomic profiles obtained from the RNA-seq analysis. As shown in Figure 6, all six genes exhibited markedly higher expression levels in tumor-derived cells compared to their patient-matched non-tumor counterparts.

*FAP* exhibited a highly specific expression pattern, with near-negligible levels in all N samples and robust upregulation across the tumor cases. This distinct expression profile suggests that FAP serves as a particularly potent candidate marker for discriminating between T and N phenotypes in our patient-derived models. Furthermore, genes associated with extracellular matrix remodeling and oncogenic signaling, such as *SPARC* and *WNT5A*, showed significant fold-changes relative to the PK-56N baseline. These results reinforce the technical accuracy of our sequencing pipeline and the biological relevance of the identified gene signatures.

### 3.8. Immunohistochemical Validation of FAP Expression

**Figure 7:**
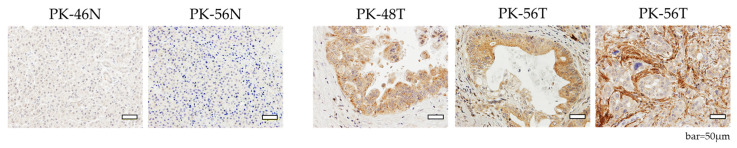
IHC analysis of FAP expression in pancreatic tissues. Representative images of FAP staining in patient-matched N (PK-46N, PK-56N) and T (PK-48T, PK-56T) sections. FAP expression is virtually absent in N tissues. In contrast, T tissues exhibit robust FAP immunoreactivity in both cancer cells and the surrounding stroma. In PK-56T, FAP-positive fibroblasts (about CAF) are scarce in areas with well-differentiated tumor morphology. However, intense FAP staining is observed in the fibroblasts (about CAF) surrounding T clusters with relatively lower differentiation, suggesting a correlation between stromal activation and T grading. Scale bars = 50 μm.

To evaluate the spatial distribution of FAP, we performed immunohistochemistry on FFPE sections from matched patient samples. In N tissues (PK-46N and PK-56N), FAP expression was virtually undetectable. In contrast, T tissues (PK-48T and PK-56T) exhibited intense FAP immunoreactivity. FAP was observed not only in the surrounding stromal fibroblasts, consistent with its role as a cancer-associated fibroblast (CAF) marker, but prominently within the pancreatic cancer cells themselves (Figure 7). This localized expression in the tumor epithelium correlates with the high levels of FAP mRNA detected by RNA-seq and qRT-PCR, suggesting that these cells may have acquired mesenchymal-like characteristics.

## 4. Discussion

In the present study, we established a robust primary culture platform for PDAC that maintains high genomic and phenotypic fidelity. The high success rate (over 90%) and rapid expansion of patient-derived cells represent a significant advancement over conventional models, providing a reliable platform for both molecular discovery and functional diagnostics within a clinically relevant timeframe (within three weeks for determining the therapeutic agent) [6].

While a direct side-by-side comparison was not performed in this study, previous reports on PDAC organoids indicate a median turnaround time of 4–12 weeks and a success rate of approximately 70–80% [6,9]. In contrast, our method achieved a 92.9% success rate and drug sensitivity results within only 2–3 weeks. This rapid processing is a critical advantage for clinical application in advanced PDAC.

Morphologically, the consistent cobblestone appearance and robust EpCAM expression across our established lines are consistent with the maintenance of epithelial identity. A key advantage of our system is its ability to culture patient-matched non-tumor and tumor cells under identical conditions. The inherent growth advantage of malignant cells facilitates efficient enrichment in the early stages of culture. Our method intentionally enriches EpCAM-positive malignant cells to evaluate the intrinsic drug sensitivity of the epithelial cancer component, which is the primary target of chemotherapy. While stromal and immune cells are excluded, this reductionist approach allows for a clearer, more reproducible readout of cancer cell response, minimizing the confounding effects of stromal overgrowth often seen in primary cultures. This allows for precise intrapatient comparative studies, effectively eliminating interindividual genetic background noise [10].

The genomic stability of our model, specifically the preservation of *KRAS* mutations through multiple passages, supports its use as a “functional proxy” for the original tumor. Furthermore, using viable cultured cells offers a superior alternative to FFPE specimens. Unlike the degraded and chemically modified nucleic acids typical of FFPE samples, our platform provides high-molecular-weight, intact DNA and RNA, ensuring high-fidelity transcriptomic data and accurate detection of complex genetic alterations [20].

Our integrative RNA-seq and qRT-PCR analysis identified a signature set of six pancreas-related genes (*FAP*, *IGFBP5*, *PRRX1*, *SPARC*, *WNT5A*, and *ADAMTS12*) consistently upregulated in tumor cells. These genes are not merely markers of malignancy; they may represent part of the core biological programs in PDAC progression.

FAP and PRRX1 (epithelial–mesenchymal transition axis): The robust expression of *FAP* and the epithelial–mesenchymal transition-related transcription factor *PRRX1* in our tumor-derived cells suggests our culture system preserves the high-plasticity state of PDAC [21]. While *FAP* is viewed as a stromal marker, its presence in the tumor epithelium, as confirmed by our IHC analysis, indicates an epithelial–mesenchymal transition program. This is evident in poorly differentiated regions, where *FAP* expression shifts from the stroma to tumor cells, potentially related to increased invasiveness and stemness [22].

SPARC and ADAMTS12 (matrix remodeling): Enrichment of *SPARC* and *ADAMTS12* shows the model’s ability to reflect the desmoplastic nature of PDAC. *SPARC* is a critical regulator of cell–matrix interactions and has been implicated in modulating drug delivery [23], while *ADAMTS12* functions in extracellular matrix degradation, linked to the physical invasion of cancer cells into surrounding tissue [24].

WNT5A and IGFBP5 (survival and resistance): Upregulation of *WNT5A* (a key ligand in non-canonical Wnt signaling) and *IGFBP5* points toward activated pro-survival and chemoresistance pathways. *WNT5A* has been reported to correlate with tumor cell motility [25], while *IGFBP5* modulates insulin growth factor signaling implicated in cell survival under therapeutic stress [26], aligning with the drug resistance profiles observed in our cohort.

The clinical relevance of our platform was demonstrated by the correlation between in vitro chemosensitivity and patient outcomes. The cases of PK-46 and PK-60 were particularly illustrative: their in vitro resistance to gemcitabine and SN38 paralleled their clinical progression and CA19-9 dynamics during NAC. This speed is critical, as the window for selecting effective therapies is often narrow [6,9]. The ability to generate such functional data rapidly (faster than typical 3D organoid models) makes this 2D adherent system a pragmatic choice for guiding personalized treatment strategies. This approach may be useful for predicting adverse events and realizing personalized medicine independent of randomized controlled trials.

The accessible monolayer nature of our culture system is uniquely suited for validating cell surface markers. This includes assessing immune checkpoint molecules like PD-L1 and identifying targets for next-generation therapies, such as antibody–drug conjugates [25]. By providing high-quality biological material and rapid functional insights, our approach provides a scalable infrastructure for advancing personalized medicine in PDAC. It aids current chemotherapeutic selection and paves the way for advanced personalized immunotherapy and targeted drug development in PDAC.

Compared to 3D organoid cultures, our 2D adherent culture platform has practical advantages. Although organoids are valuable for mimicking tissue architecture, they may require complex extracellular matrices and long growth periods (6–8 weeks), which delays clinical decision-making. Our method enables rapid expansion of patient-derived cells. Chemosensitivity screening can be completed quickly (within 3 weeks), which is critical for pancreatic cancer patients where selection of effective neoadjuvant or adjuvant therapies is urgent.

The utility of this model is reinforced by its high genomic fidelity. As demonstrated by our ddPCR results, the preservation of key *KRAS* mutations through several passages ensures cultured cells remain representative of the original tumor. This stability supports our platform as reliable, providing a consistent genetic background for both molecular profiling and drug testing.

This study had some limitations. First, it involved a small sample size (8 patients). Despite the high success rate and consistent results, a larger cohort is needed to validate the applicability of this culture platform across clinical stages and molecular subtypes of PDAC. Second, while our 2D adherent culture system effectively preserves tumor-specific molecular signatures, it lacks the complex 3D architecture and interactions with immune and stromal components found in in vivo tumor microenvironment [27,28]. This study does not clarify how the loss of non-epithelial cells affects drug sensitivity outcomes. Future studies that incorporate co-culture systems or 3D scaffolds may provide a more comprehensive understanding of these interactions. Third, although we observed tumor-derived cells eventually dominated the culture due to their growth advantage, residual normal epithelial or stromal cells could still be present in early passages. Refinement of cell sorting techniques could further enhance the purity of established cancer cell lines. Finally, while we observed a pattern suggesting a relationship between in vitro drug resistance and poor patient prognosis, the limited duration of clinical follow-up necessitates additional prospective clinical studies. Long-term, longitudinal studies are needed to determine whether drug sensitivities observed in our platform can accurately predict long-term survival and recurrence patterns in a clinical setting. Despite these limitations, the high establishment success rate demonstrated in this study and the advantage of utilizing high-quality nucleic acids are important for personalized medicine in pancreatic cancer.

## 5. Conclusions

In summary, we have developed a novel, high-success-rate adherent culture system that overcomes several practical and intellectual property-related limitations of conventional organoid models. Our findings provide preliminary evidence that in vitro drug sensitivity and transcriptomic profiling using this platform may reflect the biological characteristics of the original tumor. However, given the exploratory nature of this study and the limited sample size, these results should be interpreted as suggestive rather than confirmatory. This platform represents a promising first step toward rapid functional precision medicine, and future prospective trials are warranted to establish its definitive role in clinical decision-making.

## 6. Patents

This research is based on the following patent and PCT application technologies; Patent No. 7735022, PCT/JP2024/046241.

## Figures and Tables

**Table 1:** Summary of patient demographics and tumor characteristics of cases in this study. ID numbers including N indicate no tumor cells were detected in tissue specimens, whereas those including T indicate tumor cells were detected. The pathological stage and histological differentiation listed for non-tumor samples indicate findings from the paired tumor specimens.

Sample ID	Age/Sex	Location	Pathological Stage	Histological Differentiation	Neoadjuvant Chemotherapy
PK-40N	61/M	Head	1b	Moderate	GEM + nabPTX
PK-46N	65/M	Head	2	Moderate	GEM + TS-1
PK-53N	83/M	Head	1a	Poor	No
PK-56N	85/M	Tail	1b	Poor	No
PK-59N	82/M	Head	1a	Moderate	No
PK-48T	79/F	Body	1a	N/A	GEM + TS-1
PK-52T	77/M	Head	1a	Moderate	No
PK-56T	85/M	Tail	1b	Poor	No
PK-60T	46/M	Body	1a	Moderate	GEM + nabPTX

**Table 2:** Genes analyzed by qRT-PCR and their TaqMan™ Primer & Probe sets.

*Gene Name*	*Gene Symbol*	Assay ID
*F* *ibroblast activation protein alpha*	*FAP*	Hs00990791_m1
*Insulin-like* *growth factor binding protein 5*	*IGFBP5*	Hs00181213_m1
*P* *aired related homeobox 1*	*PRRX1*	Hs00246567_m1
*S* *ecreted protein acidic and cysteine rich*	*SPARC*	Hs00234160_m1
*Wnt family member 5A*	*WNT5A*	Hs00998537_m1
*ADAM metallopeptidase with thrombospondin type 1 motif 12*	*ADAMTS12*	Hs00917098_m1
*Actin beta*	*ACTB*	Hs99999903_m1

**Table 3:** Drug sensitivity profiles and clinical outcomes of patient-derived pancreatic cancer cells.

Sample ID	Gemcitabine (µM)	Paclitaxel (nM)	SN38 (nM)	Early Recurrence	Adverse Events
PK-40N	5 (>100) ^1^	50 (>100)	n.d.	4 months	No
PK-46N	0.1 (0.1) ^1^	500 (50)	10 (10)	6 months	Oral ulcer
PK-53N	5 (>100) ^1^	50 (>100)	50 (1000)	3 months	No
PK-56N	1 (>100) ^1^	10 (50)	10 (>1000)	1.5 months	No
PK-48T	5	500	50	No	No
PK-52T	0.5	>1000	50	No ^2^	No
PK-56T	>100	500	>1000	6 months	No
PK-60T	>100	>1000	500	No	Interstitial pneumonia

^1^ Values in parentheses for N samples indicate the *EC*_50_ for cells derived from the tumor region of the same case. ^2^ Multiple cancers in organs other than the pancreas.

## Data Availability

The original contributions presented in this study are included in the article/Supplementary Material. Further inquiries can be directed to the corresponding author.

## Supplementary Materials

The following supporting information can be downloaded at: https://www.mdpi.com/article/10.3390/cells15040313/s1, Table S1: Information regarding the sequence of primers and probes used in ddPCR; Table S2: Results of Gene Ontology and KEGG pathway analyses; Figure S1: Volcano plots illustrating differentially expressed genes in (a) a representative patient-matched pair (PK-56N vs. PK-56T) and (b) all pooled N vs. T samples (PK-All(N) vs. PK-All(T)).

## Abbreviations

The following abbreviations are used in this manuscript:

ACTB	Actin beta
ADAMTS12	ADAM metallopeptidase with thrombospondin type 1 motif 12
CAF	Cancer-associated fibroblast
ddPCR	droplet digital polymerase chain reaction
DEGs	Differentially expressed genes
EpCAM	Epithelial Cell Adhesion Molecule
FAP	Fibroblast Activation Protein Alpha
FFPE	Formalin-fixed paraffin-embedded
GO	Gene ontology
IGFBP5	Insulin-like growth factor binding protein 5
IHC	Immunohistochemical
N	Non-tumor
NAC	Neoadjuvant chemotherapy
PDAC	Pancreatic ductal adenocarcinoma
PRRX1	Paired related homeobox 1
qRT-PCR	quantitative reverse transcription- polymerase chain reaction
RNA-seq	RNA sequencing
SPARC	Secreted protein acidic and cysteine rich
T	Tumor
WNT5A	Wnt family member 5A
