# Supplementary figures and images for: Rapid Drug Sensitivity Profiling via a Novel High-Success-Rate Culture Method for Patient-Derived Pancreatic Cancer: An Exploratory Preclinical Platform for Advancing Clinical Applications and Drug Development
Source: Cells. 2026 Feb 7;15(4):313. doi: 10.3390/cells15040313 (PMC12939418; doi:10.3390/cells15040313)

## Slide 1
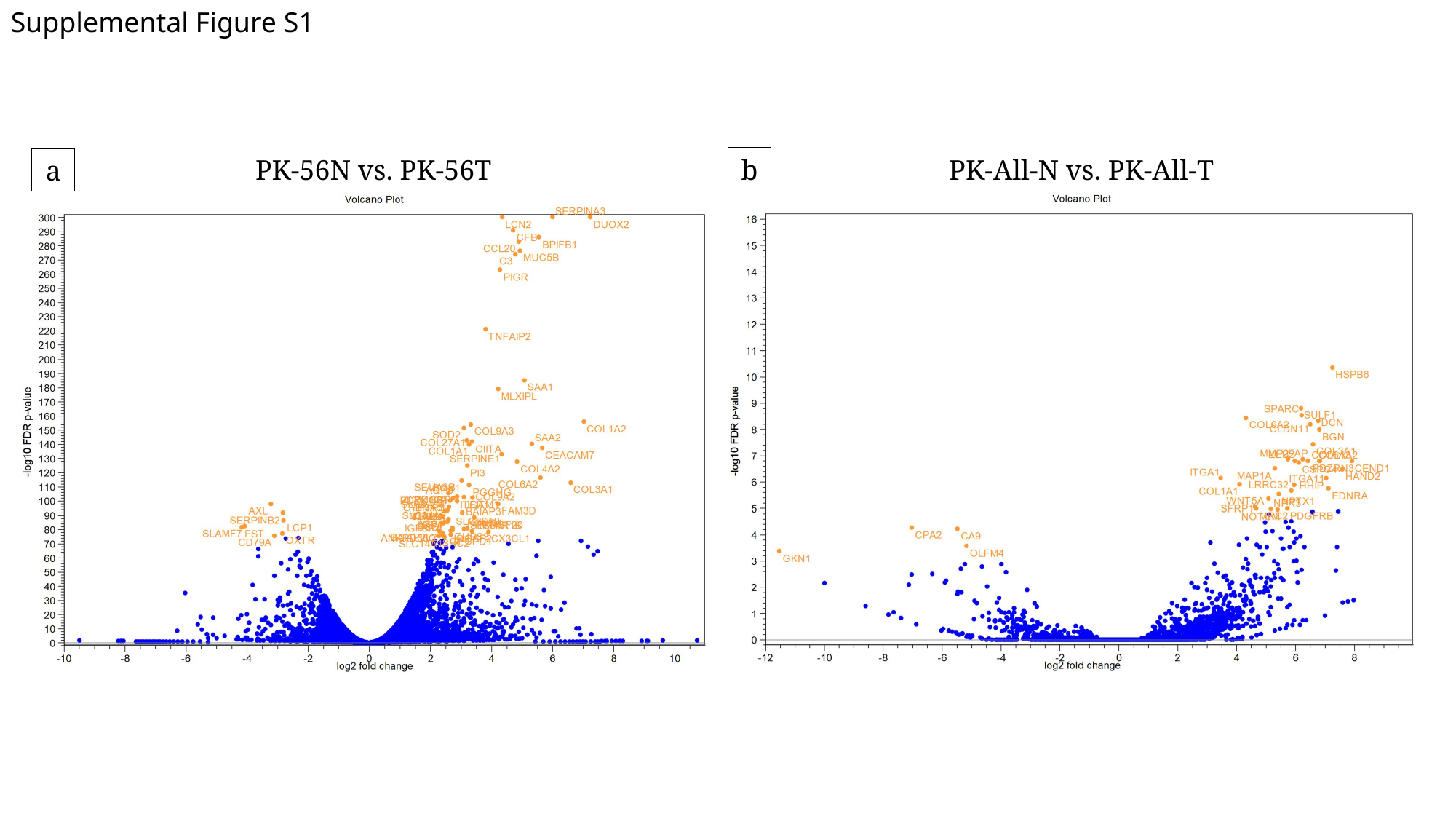

Supplemental Figure S1
PK-56N vs. PK-56T
b
PK-All-N vs. PK-All-T
a

Supplement: Supplementary file 1 [file cells-15-00313-s001.zip › Supplemental Figure S1.pptx]
